# Comparative study on preoperative localization techniques using microcoil and hookwire by propensity score matching

**DOI:** 10.1111/1759-7714.13365

**Published:** 2020-03-24

**Authors:** Feng Yang, Hui Zhao, Xizhao Sui, Xugang Zhang, Zewen Sun, Xiaoyi Zhao, Jun Wang

**Affiliations:** ^1^ Department of Thoracic Surgery, Center for Mini‐invasive Thoracic Surgery, People's Hospital Peking University Beijing China; ^2^ Department of Thoracic Surgery Shijitan Hospital Beijing China

**Keywords:** Localization, lung cancer, nodule, thoracoscopic

## Abstract

**Background:**

The purpose of this study was to compare the efficacy and safety of two preoperative pulmonary nodule localization techniques using microcoil and hookwire.

**Methods:**

A total of 307 patients with 324 pulmonary nodules were included in the study from March 2012 to October 2016 in two medical centers. Baseline data, positioning operation data, success rate, complications, surgery and pathological results were statistically analyzed. Complications were used as the dependent variables, whereas others were used as covariates for the propensity score matching of the two groups. Statistical analyses were performed to compare the success rate and complication rate of the matched groups.

**Results:**

There were 218 lesions in the microcoil group and 106 nodules in the hookwire group. There were no significant differences in gender, age and the location of nodules between the two groups. The diameters of the nodules were smaller (8.2 ± 3.5 mm vs. 10.7 ± 4.3 mm) and solid nodules were fewer (11.5% vs. 26.4%) in the microcoil group. The complication rate of the two groups was not statistically significant. After propensity score matching, 71 patients in each group were successfully matched. We found that the success rate was higher (97.2% vs. 94.4%) and the incidence of complications was lower (31% vs. 15.5%) in the microcoil group.

**Conclusions:**

Both techniques have been shown to be effective in preoperative localization of tiny pulmonary nodules. The method of microcoil localization has more advantages in clinical application.

**Key points:**

Comparison of the efficacy and safety of two methods in preoperative pulmonary nodule localization in order to determine the optimal method.

## Introduction

With the increasing use of low‐dose computed tomography (LDCT) imaging in clinical practice and lung cancer screening, solitary pulmonary nodules (SPNs) are being detected more frequently.^1^ The standard treatment for potential malignant SPNs is complete resection by video‐assisted thoracic surgery (VATS). However, some SPNs, for example subcentimeter nodules or ground‐glass nodules, are often not visible or palpable during surgery due to their small size and soft texture, making it impossible for surgeons to detect intraoperatively. Thus, preoperative localization of these nodules is very helpful for guiding resection through VATS. There are a substantial amount of localization tools including hookwire, microcoil, lipiodol, barium, radionuclides, and dye materials. Among the localization techniques, hookwire and microcoil stand out as the two most widely used methods owing to their convenience. The majority of previous studies investigating localization techniques focus on a single method. Few comparisons have been made between different methods regarding their effectiveness and safety. In 2016, Park *et al*. evaluated the best VATS localization method involving hookwire, microcoil and lipiodol by analyzing data from 46 clinical studies.^2^ The results showed that all three localization methods yielded similarly highly successful targeting rates. However, hookwire localization had a relatively lower success rate because of dislocation or migration, while microcoil localization had the lowest complication rate. Although this study carried out a high quality pooled analysis, noncomparative baseline information existed between different the groups because the majority of enrolled studies were observational studies that incorporated only one type of localization method. Thus, more studies are still needed to find the optimal localization method. In this study, we compare the effectiveness and safety of two localization methods involving hookwire localization and microcoil localization in propensity‐score matched cohorts from two different institutions.

## Methods

### Research subjects

Data were obtained from two medical centers; Peking University People's Hospital and Beijing Shijitan Hospital. A total of 205 patients were recruited from December 2012 to April 2015 into the microcoil group, and 102 patients were recruited from March 2012 to October 2016 into the hookwire group. The use of the microcoil and hookwire was approved by the Institutional Review Board of Peking University People's Hospital (IRB No.2014PHB113‐01). Written informed consent forms were signed by all patients.

### Inclusion criteria

The inclusion criteria for the two groups were as follows: (i) Solid nodules ≤10 mm in diameter that were ≥ 5 mm from the visceral pleura, (ii) pure ground‐glass lesions, (iii) mixed ground‐glass lesions with solid composition ≤10 mm in diameter and ≥10 mm from the pleura, (iv) lesions without pleural indentation not involving the pleura, and (v) deep pure ground‐glass lesions with an easily accessible location for cutting.

The exclusion criteria for the two groups was basically the same: (i) Nodular diameter >30 mm or obvious pleural depression easy to locate during surgery, (ii) patient previously having suffered from tuberculous pleurisy, or imaging had revealed extensive pleural adhesions thus making it impracticable for VATS, (iii) inability to puncture the patient, and (iv) nodules located in one third of the lung field or near the hippocampal blood vessels or bronchus and obscured from view.

### Puncture equipment

**Figure 1:**
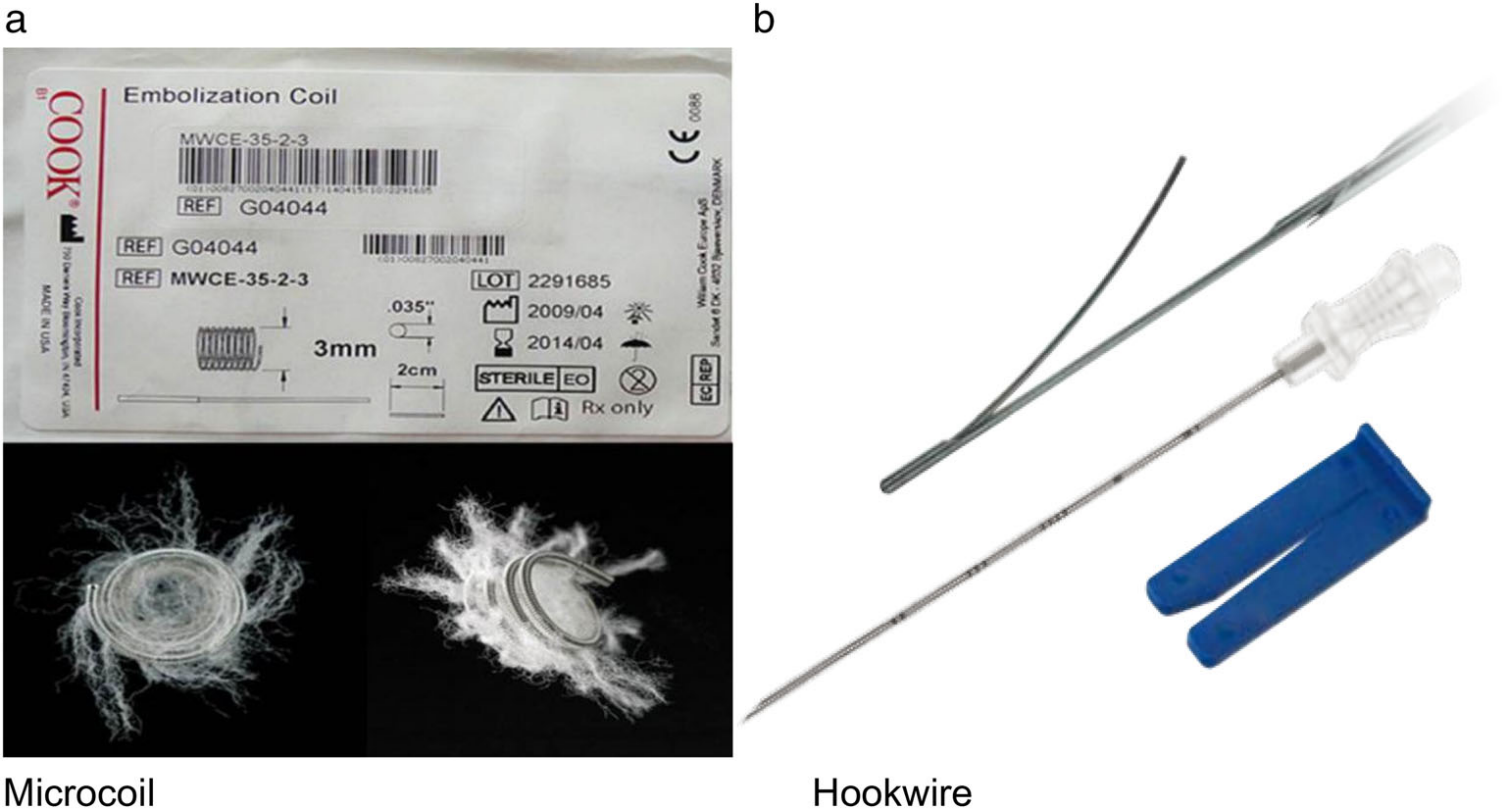
(**a**) Cook's embolization microcoil. (**b**) Angiotech's hookwire.

Cook's embolization microcoil (Cook Inc., Bloomington, IN, USA) was used in the study, consisting of a platinum‐based alloy with artificial nylon fibers on the surface, which activate the clotting complex and promote thrombosis. The microcoil had a wire length of 6–12 cm and a microcoil spiral diameter of 3 mm (Fig 1(a)). The hookwire positioning pin used in this study was Accura‐BLN2110 breast positioning needle manufactured by Angiotech (USA) (Fig 1(b)); the inner wire was stainless steel and approximately 30 cm in length, hard, flexible, and easy to restore after bending. A 21 G puncture trocar was used in both groups which was 10 cm in length.

### Procedure

**Figure 2:**
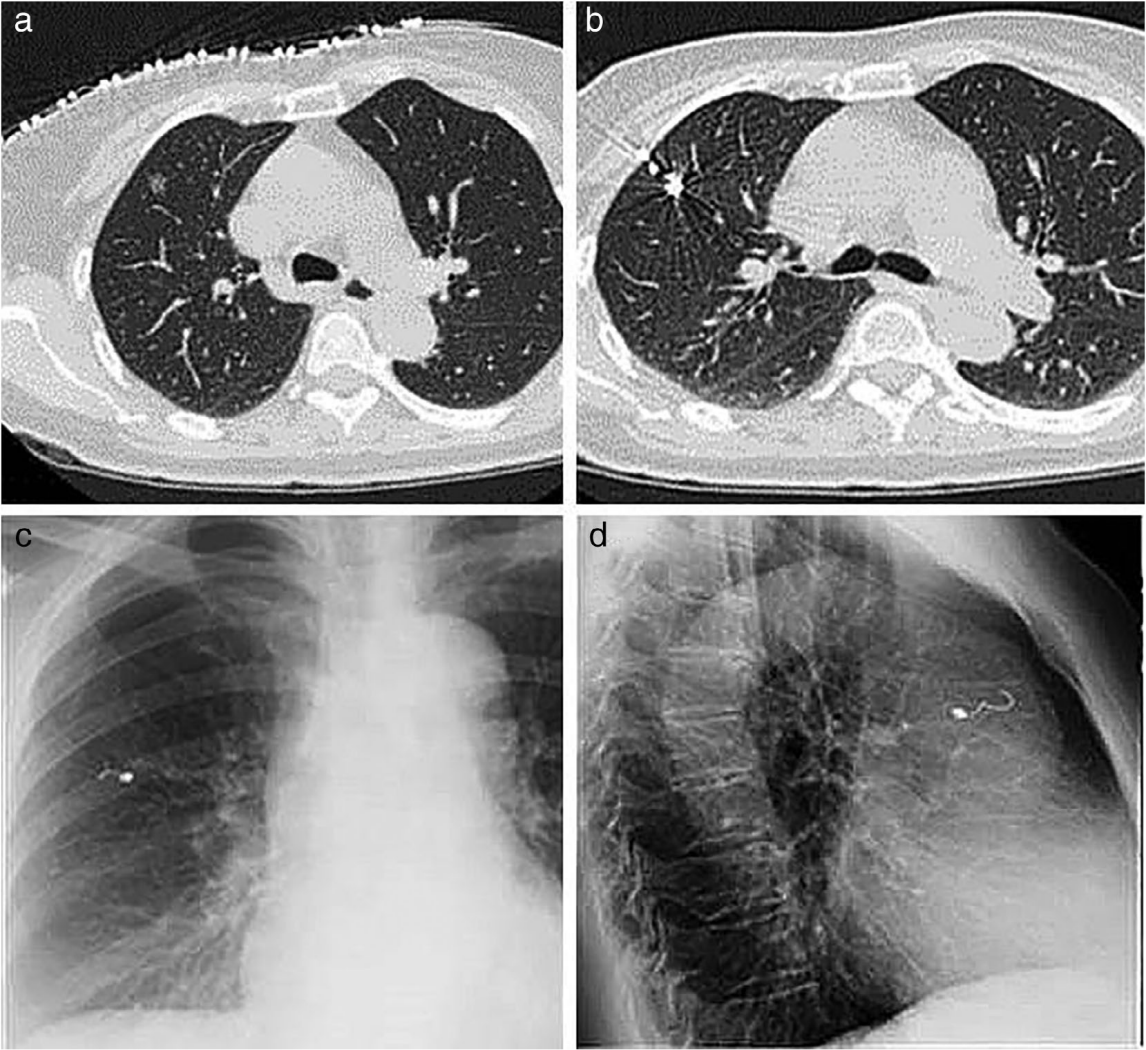
Imaging findings before and after coil positioning operation. (**a**) CT scan before localization found pulmonary nodules. (**b**) CT scan during localization confirmed that the puncture need lewas located in the lung tissue around the lesion. (**c**, **d**) chest X‐ray after localization showed the proximal part coiling beyond the parietal pleura and the distal part anchoring in the lung parenchyma.

The operators of the localization procedure in both of the two hospitals were board‐certified interventional radiologists with 3–5 years' experience. Three operators were involved in the study. Patients in the microcoil group were referred to the Department of Radiology for preoperative localization on the day of surgery or 1–2 days before the surgery. First, according to the preoperative CT data, the location of the lesion and puncture path were determined avoiding the ribs, scapula, large blood vessels, fissure of the lung and lung bullae. The location of the needle, edge of the lesion and vertical distance between the skin and the parietal pleura were measured using a three‐dimensional reconstruction technique to simulate the puncture path. Following conventional disinfection, placement of surgical draping and application of 2% lidocaine local anesthesia, a 21 G trocar percutaneous puncture needle was utilized, advancing the needle tip into the deep peripheral lesions of the normal lung parenchyma. Another CT scan was then performed to confirm that the puncture needle was located in the lung tissue around the lesion, and then the microcoil core and puncture needle were connected using the “conventional method” or “tailing method” (Fig 2). After successfully completing this procedure, the patient returned to the ward and was advised to try to avoid coughing. On the day of surgery, chest X‐rays were not routinely performed on patients without any symptoms; on the day after surgery, a chest X‐ray was performed in the morning to determine whether any complications had occurred.

**Figure 3:**
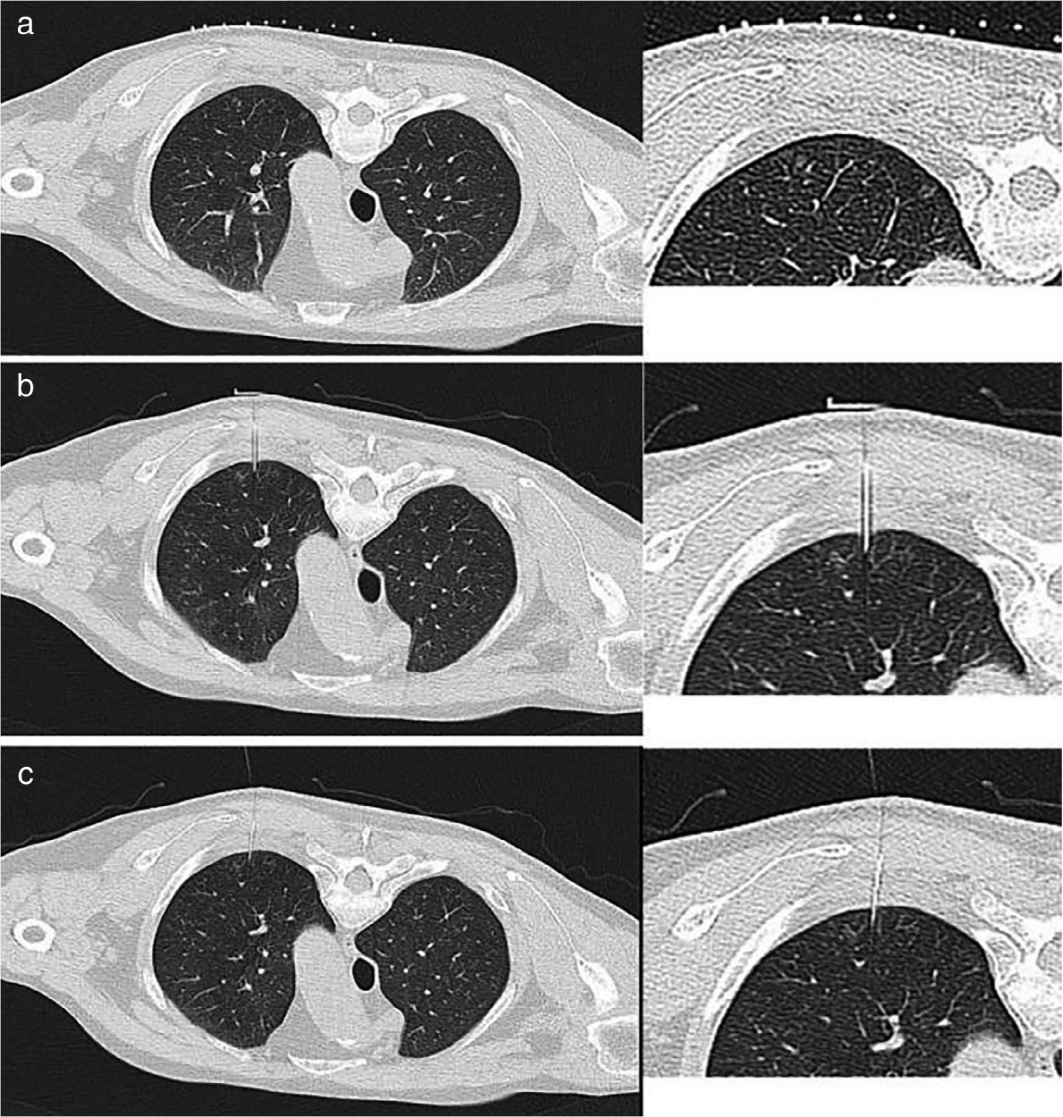
The positioning process of hookwire with CT guided. CT scan chose puncture plane before operation (**a**), CT scan determines the angle and depth of insertion during operation (**b**), and CT scan confirmed that the hookwire was located in the lung tissue around the lesion after operation (**c**).

Patients in the hookwire group were sent to the CT room one hour before surgery for hookwire puncture positioning. The positioning process was similar to that described for the microcoil group (Fig 3). A 2 cm incision was made in the skin at the location of wire positioning, gauze was used to cover the wound, and the patient was immediately sent to the operating theater.

Four experienced board‐certified thoracic surgeons who majored in video‐assisted thoracoscopic surgery (VATS) were involved in the study. Conventional thoracoscopic surgery was performed. The patients were on a ventilator with a double lumen endotracheal tube and placed under general anesthesia in a lateral position with ipsilateral one‐lung ventilation. Two to three ports comprising observation, main operating and/or auxiliary ports were made. The observation port was located at the midaxillary line of the seventh or eighth rib. The main operating port was located at the anterior axillary line of the fourth or fifth rib with a length of 3–4 cm, while the auxiliary incision was made at the infrascapular line of the seventh or eighth rib. During VATS surgery, the microcoil group was examined at the trailing end of the microcoil to determine the location of the lesion. If the microcoil was not located, fluoroscopy was utilized to find the microcoil and the integrity of the coil was confirmed. Both groups underwent wedge resection of the lesions, and after surgery the lesions were subjected to intraoperative frozen section examination. Pathological results included benign lesions, atypical adenomatous hyperplasia (AAH) and secondary pulmonary cancer. Based on the results of frozen section diagnosis, the operative procedure ended for patients with benign lesions or noninvasive lung cancer. Patients with invasive lung cancer subsequently underwent thoracoscopic lobectomy and lymph node dissection or sampling. Patients with suspected invasive lung cancer underwent lobectomy or sublobar resection and lymph node sampling following their preoperative willing informed consent.

### Statistical analysis

Data were analyzed using IBM SPSS 22.0 and R software (PS matching module). The propensity score matching function was used to match the two groups of patients with complications as the dependent variable and sex, age, nodule density, nodule diameter, nodule to pleural distance and nodular position were the covariates in the logistic regression. The tendency score was derived using the 1:1 nearest neighbor matching method, and the caliper value was set to 0.05 to ensure optimal matching. The matched samples were tested for intergenerational covariance. Statistical analyses were performed using the matched groups. The data were compared with the χ^2^ test or Fisher's exact test. The data were expressed as x ± s, and the *t*‐test was used. Normally distributed data and variables were not analyzed using the Mann‐Whitney test. *P* < 0.05 indicated a statistically significant difference.

## Results

### Comparison of baseline data between groups of patients

A total of 307 patients were recruited into the study including 205 patients in the microcoil group and 102 patients in the hookwire group (see Table 1). There were significant differences in nodular characteristics between the two groups: the hookwire group exhibited larger nodule diameters and a greater number of solid nodules than the microcoil group, and the difference was statistically significant (*P* < 0.05). The distance between the nodule and pleura was also greater in the hookwire group than the microcoil group, but the difference was not statistically significant. The operation time in the microcoil group was 19.3 ± 4.5 minutes, and the interval between positioning success and the beginning of the operation was 15.8 ± 12.4 hours, which was significantly longer than that in the hookwire group. Due to the “trailing method” used in the microcoil group, the operation was relatively complex, which resulted in a longer operative procedure. Moreover, while the microcoil group underwent surgery on the day of positioning or 1–2 days after positioning, the hookwire group immediately underwent surgery after positioning; thus, the interval between positioning and the surgery in the microcoil group was longer. However, the depth of the needle in the hookwire group was greater than that in the microcoil group (*P* < 0.05).

**Table 1:** Patient characteristics

	Microcoil	Hookwire		
Patient characteristics	*N* = 205	*N* = 102	χ2 value or t value	*P*‐value
Age	55.8 ± 10.7	57.9 ± 11.3	1.619	0.106
Gender			2.920	0.088
Male	68 (33.2%)	44 (43.1%)		
Female	137 (66.8%)	58 (56.9%)		
Nodular density			11.65	0.001
Solid	25 (11.5%)	28 (26.4%)		
Nonsolid	193 (88.5%)	78 (73.6%)		
Nodular diameter (mm)	8.2 ± 3.5	10.7 ± 4.3	5.717	0.000
Nodule to pleural distance (mm)	11.4 ± 7.4	12.9 ± 5.8	1.880	0.061
Nodule location			5.296	0.258
RUL	82 (37.6%)	35 (33%)		
RML	10 (4.6%)	12 (11.3%)		
RLL	37 (17%)	18 (17%)		
LUL	44 (20.2%)	21 (19.8%)		
LLL	45 (20.6%)	20 (18.4%)		
Operation time (minutes)	19.3 ± 4.5	14.3 ± 5.3	8.519	0.000
Interval time (hours)	15.8 ± 12.4	1.09 ± 0.2	11.961	0.000
Depth of needle (mm)	11.9 ± 7.1	21.9 ± 8.2	11.267	0.000
Double localization	13 (6.3%)	4 (3.9%)	0.762	0.383

### Comparison of complications and dislocation in both groups

The complication rate was 19% (39/205) in the microcoil group and 26.5% (27/102) in the hookwire group, but the difference was not statistically significant (*P* > 0.05). Detailed data on the complications and dislocations are shown in Table 2.

**Table 2:** Incidence of complications and dislocations in the two groups

	Microcoil	Hookwire		
Location‐related data	*N* = 205	*N* = 102	χ2 value	*P*‐value
Complication	39 (19%)	27 (26.5%)	2.238	0.135
Pneumothorax	27 (13.2%)	15 (14.7%)		
Pulmonary hemorrhage	11 (5.4%)	7 (6.9%)		
Pneumothorax and pulmonary hemorrhage	0	4 (3.9%)		
Hemoptysis and pain	1 (0.5%)	1 (1.0%)		
Dislocation	5 (2.3%)	5 (4.7%)	1.311	0.252
Success rate	97.7%	95.3%		

### Comparison of the pathological results in both groups

There were 21 patients with benign nodules, 22 with AAH and 175 patients with malignant lesions in the microcoil group. Among the 106 nodules in the hookwire group, 33 were benign, 10 were AAH and 63 were malignant lesions. The specific pathological types are shown in Table 3.

**Table 3:** Pathological results in the two groups

	Microcoil	Hookwire
Pathology	*N* = 218	*N* = 106
Benign lesion	21	33
Inflammatory lesions	6	15
Hamartoma	2	5
Sclerosing hemangioma	0	3
Fibrous tissue hyperplasia	4	4
Lymph node lesion	9	5
Granuloma	0	1
Atypical adenomatous hyperplasia	22	10
Malignant lesion	175	63
Adenocarcinoma in situ	63	20
Microinvasive adenocarcinoma	47	15
Invasive adenocarcinoma	60	24
Squamous cell carcinoma	0	2
Metastatic carcinoma	5	2

### Comparison of tendency scores

**Figure 4:**
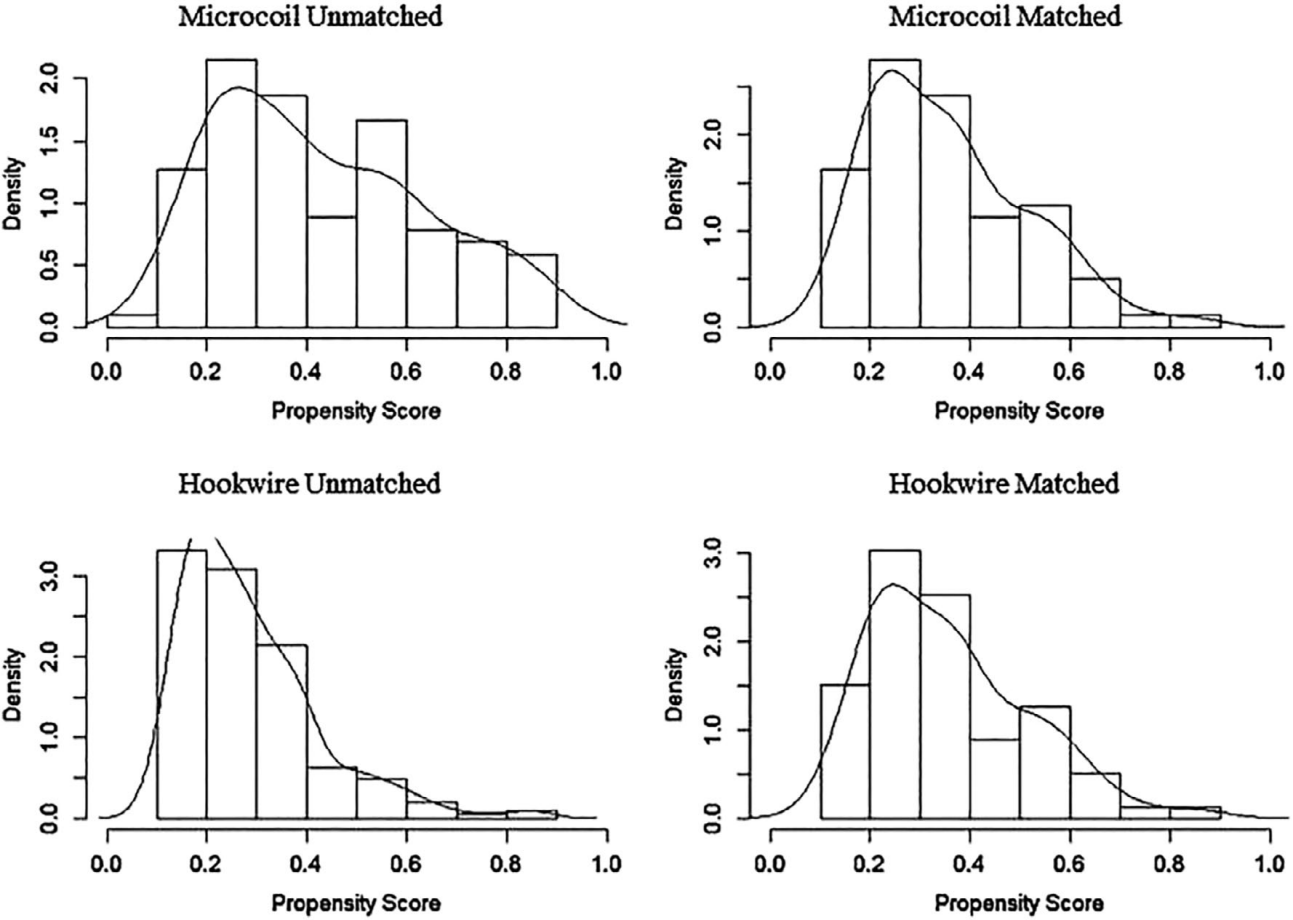
Tendency score distribution histogram. (**a** and **c**) PS histograms of the microcoil group and (**b** and **d**) the hookwire group. After matching (**c** and **d**), the histograms of the PS distributions in the two groups were similar, suggesting a good match.

Using the R matching software (PS matching module) of SPSS 22.0, the preference score was determined in the two groups. The specific steps were as follows: (i) To determine the tendency score model, the propensity score (PS) was calculated using logistic regression; the complications were the dependent variables, and sex, age, nodule density, nodule diameter, nodule to pleural distance, and the node position were covariates. (ii) Tendency score matching was performed using the 1:1 nearest neighbor matching method with the caliper value set to 0.05; thus, individuals with similar PS scores were matched with each other. (iii) The matched samples were tested for covariate equilibrium. The PS distribution histograms in Fig 4 showed similar distribution patterns in the two groups after matching, thus suggesting a good match. (iv) The matched groups were used for statistical analysis.

### Comparisons of matched groups

A total of 71 pairs were successfully matched in the two groups (see Table 4); after matching, there were no significant differences in gender, age, nodular diameter, nodule density, nodule to pleural distance and nodular baseline parameters between the two groups (*P* > 0.05). The complication rate of the microcoil group was significantly lower than that of the hookwire group (31%, *P* = 0.029). The location success rate of the hookwire group (94.4%) was lower than that of the microcoil group (97.2%), but the difference was not statistically significant (*P* > 0.05).

**Table 4:** Comparison of the scores of the two groups after matching

	Microcoil	Hookwire		
Variable	*N* = 71	*N* = 71	χ^2^ value or t value	*P*‐value
Age	55.4 ± 10.3	56.4 ± 11.5	0.524	0.601
Sex			0.123	0.725
Male	24 (33.8%)	26 (36.6%)		
Female	47 (66.2%)	45 (63.4%)		
Nodular diameter (mm)	9.3 ± 3.7	9.4 ± 3.2	0.170	0.865
Nodule to pleural distance	12.3 ± 7.2	12.3 ± 5.4	0.013	0.990
Nodule type			0.061	0.805
Solid	10 (14.1%)	9 (12.7%)		
Nonsolid	61 (85.9%)	62 (87.3%)		
Nodule location			3.682	0.451
RUL	22 (31.0%)	24 (33.8%)		
RML	4 (5.6%)	9 (12.7%)		
RLL	10 (14.1%)	12 (16.9%)		
LUL	18 (25.4%)	12 (16.9%)		
LLL	17 (23.9%)	14 (19.7%)		
Depth of the needle (mm)	12.7 ± 6.8	21.8 ± 8.5	7.028	0.00
Operation time (min)	18.8 ± 4.7	14.9 ± 5.9	4.337	0.00
Interval time (hours)	17.7 ± 14.2	1.1 ± 0.3	9.796	0.00
Complication	11 (15.5%)	22 (31.0%)	4.777	0.029
Pneumothorax	7 (9.9%)	12 (16.9%)		
Pulmonary hemorrhage	4 (5.6%)	6 (8.5%)		
Pneumothorax and pulmonary hemorrhage	0	3 (4.2%)		
Hemoptysis and pain	0	1 (1.4%)		
Dislocation	2 (2.8%)	4 (5.6%)	0.696	0.404
Success rate	97.2%	94.4%		

## Discussion

As SPNs are small and soft, subcentimeter nodules are difficult to find by touch, and without preoperative localization, complete and accurate resection by thoracoscopic surgery is very difficult.^3^ Since the initial report describing the high accuracy of hookwire localization for VATS was published in 1992 by Plunkett *et al*. a variety of preoperative localization techniques involving hookwire, microcoil, lipiodol, barium, radionuclides, and dye materials has been well established. All these methodologies have their own advantages, some of which have critical limitations that would restrict their generalized use. For example, radiation exposure during surgery is the major weakness of lipiodol or barium localization. Dye staining using methylene blue tends to diffuse easily and thus leads to over‐resection of the lung tissue. Special equipment and radio‐protection are needed when radionuclide localization is performed. In addition, injection of localization agents may provoke an inflammatory response that could impact on the final pathological diagnosis. Thus, preoperative CT‐guided microcoil and hookwire localization are becoming the two most widely used technologies in the clinic and the reported literature. Our research data showed that the success rate was 97.2% (69/71) in the microcoil group and 94.4% (67/71) in the hookwire group, and the difference was not statistically significant (*P* = 0.404), indicating that the microcoil and hookwire are effective localization technologies. A meta‐analysis^3^ in 2006 screened the English literature from 1992 to 2016, including 2365 patients who underwent hookwire localization and 459 patients who underwent microcoil localization; the success rates of the two localization methods were 98% (95% CI: 0.97–0.99) and 98% (95% CI: 0.96–0.99), respectively, and the success rates of VATS resection were 96% (95% CI: 0.94–0.97) and 97% (95% CI: 0.94–0.99), respectively.

Thoracic surgeons use the preoperative localization technique to mark the tiny nodules in the lungs. The main concern is that the marker will be displaced or shed, thus preventing the lesion from being located accurately and leading to open thoracotomy or surgical failure. The incidence of hookwire dislocation reported in the literature is between 0.4% and 21.8%,^4^, ^5^, ^6^ while the microcoil has a low dislocation rate of 0–6.7%.^7^, ^8^ As the hookwire is composed of stainless steel, it is hard and flexible, but the recovery is poor, and the barbed needle is prone to falling off the lung tissue. Microcoil is composed of platinum material, which has a soft texture; a puncture needle is advanced into the lung tissue, and after the introduction of the microcoil, the curved plate is anchored in the lung parenchyma and firmly fixed. The chest protects the wire, and neither chest wall muscle or bone activity allow the microcoil to slip out. Our data showed a microcoil dislocation rate of 2.8% and a slightly higher hookwire decoupling rate of 5.6%. Fortunately, only 10 cases of dislocation occurred among the two groups of patients, and intraoperative combined with preoperative imaging data were used to locate the local visceral subpleural puncture site or hemorrhage, and intraoperative anatomical positioning and finger palpation was performed to determine nodular position. The lesions were successfully resected without conversion to thoracotomy; thus, the lesion removal success rate by VATS was 100%. In this study, the depth of the hookwire group was significantly greater than that of the microcoil group (21.8 + 8.5 mm vs. 12.7 + 6.8 mm, *P* < 0.05). The operator of the hookwire group considered that deep wire fixation was more reliable. However, although the depth of the needle was significantly greater in the hookwire group than the microcoil group, the dislocation rate was still higher in the hookwire group than the microcoil group (5.6% vs. 2.8%), further indicating that the barbed hookwire is more prone to dislocation. In addition, increased depth of the needle leads to more damage to lung tissue, thus increasing the probability of damage to the lungs and bronchioles and leading to pneumothorax, intracerebral hemorrhage, and a greater risk of complications, such as gas embolism. In combination with the literature,^9^, ^10^ we hypothesize that the reasons underlying hookwire decoupling are as follows: (i) If the puncture needle is too superficial, it may cause insufficient strong fixation of the wire in the lung tissue; (ii) if the wire barbs on the retractable needle fail to fully extend, the friction between the puncture needle and lung tissue is decreased; (iii) improper puncture point selection which allows bone or muscle activity to affect the wire; (iv) excessive arm and shoulder activities or large size of the patient results in wire dislocation; or after general lung ventilation, the lung tissue providing traction to the wire is displaced, resulting in wire dislocation. Due to the possible scenarios described above, the scope of the application of hookwire is limited, and successful positioning must be carried out after surgery.

There were significant differences between the inclusion criteria of the two groups, and a significant difference in the baseline data between the nodules used to control the confounding factors. Nevertheless, the incidence of complications in the hookwire group was 31% (22/71), which was significantly higher than the 15.5% (11/71) complication rate in the microcoil group, indicating that despite similar characteristics of the pulmonary nodules, the incidence of complications was higher in the hookwire positioning group than in the microcoil positioning group. In addition, because patients in the hookwire group underwent surgery immediately after hookwire placement, no imaging examinations were performed to monitor the incidence of complications; thus, the presence of complications was based only on the last CT scan, signifying that the actual complication rate in the hookwire group may be higher. CT‐guided fine needle aspiration biopsy‐related complications are as follows: pneumothorax, perirenal hematoma, hemothorax, pain, air embolism and needle tumor implantation as well as air embolism and tumor implantation, which are extremely rare, with an incidence rate of 0.061% and 0.012–0.061%, respectively.^11^ However, CT‐guided microcoil or hookwire positioning is different from puncture biopsy as the needle is positioned without repeated puncture, and the tumor is not punctured as the needle is placed in the upper and lower edges of the tumor for surgical resection after labeling; thus, the incidence of complications is low. Moreover, there is no needle transfer. Postoperative complications include pneumothorax, intracerebral hemorrhage, hemoptysis and hemothorax. For thoracoscopic surgery, the thoracic puncture can be positioned at the site of lung tissue puncture or at sites of bleeding; thus, most of the complications do not need to be addressed here. In this study, there was one case of severe chest pain in the hookwire group which was treated with intramuscular analgesia. In the microcoil group,two patients had complications that required treatment, one for medium‐sized pneumothorax on the day after positioning, the other for thoracic puncture wound. Most of the complications reported in the literature are asymptomatic, and postoperative surgical treatment addresses complications at the same time, requiring fewer cases of clinical intervention, which have previously been reported to occur in approximately 0%–4.6% of cases.^12^, ^13^, ^14^

For lesions at certain locations, such as those in front of the scapula, choosing the hookwire positioning method may lead to failure or induced disease from scapula, latissimus dorsi, or chest wall muscle displacement. If microcoil positioning method is chosen, the patient's activities after successful positioning will not affect the microcoil. Therefore, we recommend selecting the microcoil method for tumors in front of the scapula. In addition, the positioning methods for double lesions are more difficult than those for single lesions. The operation process is long, as positioning the second nodule requires additional time; moreover, the puncture path must be redesigned, and patients must change positions. Thus, positioning for double lesions has a higher complication rate than positioning for single lesions. In the case of hookwire positioning, the steel wire tail is placed in the pleural wall; thus, the patient's muscle activity can easily affect the location of the wire. Positioning of the second nodule also requires a change in body position, disinfection, anesthesia, puncture and other processes, which may interfere with the first nodular positioning of the wire; thus, pneumothorax or decoupling can easily occur. Moreover, a pneumothorax may directly affect the positioning accuracy of the second nodule. In this study, a total of 17 patients required double positioning markers to locate the pulmonary nodules, of which 13 patients in the microcoil group received double microcoil positioning markers, and four patients experienced complications (30.8%); in the hookwire group, four patients received double positioning markers, and three patients experienced complications, leading to a high complication rate of 75%. Therefore, when a patient has two or more lesions that require positioning, microcoil positioning is recommended.

It is important to consider our study in the context of its limitations. First, because of the retrospective methods of our analysis, patients were not randomized, and selection bias was inevitable. To improve the accuracy of the data, the propensity score matching function was used, reducing the effect of age, gender, nodule density and other factors on the success and complication rates. Second, the time length between location and operation varied, and some complications may be masked by a shorter interval time between localization and surgery in the hookwire group; however, this does not conflict with the final conclusions of the study.

Despite these limitations, our study provides new insights into preoperative localization techniques. In summary, the hookwire positioning technique is simple and does not require intraoperative perspective, but positioning must be carried out immediately before surgery to prevent the wire from shifting or falling off, which requires coordination among thoracic surgeons, radiologists, theater technicians and other departments. The microcoil technique has notable advantages compared to the hookwire technique. (i) It consists of platinum alloy material which has a soft texture, causes less damage to lung tissue and is reliable, with lower rates of complications and decoupling; (ii) lack of wire outside the chest wall reduces the need to restrict patient activities, and the operation time is flexible; (iii) the puncture angle is more flexible and is suitable for lesions in front of the scapula as well as small nodules at varying depths; and (iv) it is more suitable for dual or multiple lesion positioning. Therefore, evaluation of the microcoil in a wider range of clinical applications in the future should be carried out.

## Disclosure

No authors report any conflict of interest.

## Supporting information


**Figure S1** Cook's embolization microcoil.

**Figure S2** Angiotech's hookwire.
