# Supplementary figures and images for: Comparative study on preoperative localization techniques using microcoil and hookwire by propensity score matching
Source: Thorac Cancer. 2020 Mar 24;11(6):1386–95. doi: 10.1111/1759-7714.13365 (PMC7262885; doi:10.1111/1759-7714.13365)

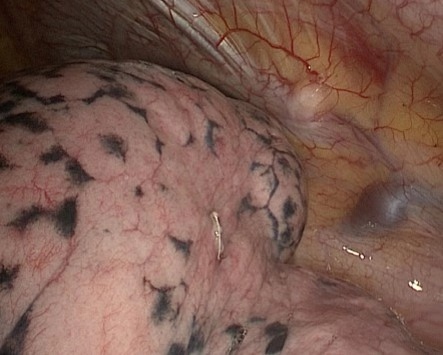

Supplement: Supplementary file 1 — Figure S1 Cook's embolization microcoil. [file TCA-11-1386-s001.jpg]

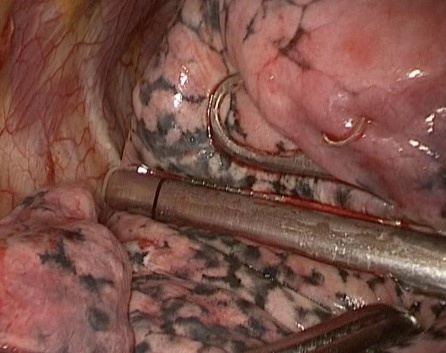

Supplement: Supplementary file 2 — Figure S2 Angiotech's hookwire. [file TCA-11-1386-s002.jpg]
